# Pathological variations in mummified feet between two near-distance/long-time populations in Ancient Egypt

**DOI:** 10.1186/s13047-015-0115-4

**Published:** 2015-10-26

**Authors:** Albert Isidro, Beatrice Huber, Aamer Malik, Assumpció Malgosa

**Affiliations:** Hospital Universitari del SagratCor, Barcelona, Spain; Äegyptolisches Institut, Eberhart-Karls University, Tübingen, Germany; Hospital Universitari Sagrat Cor, Barcelona, Spain; Department Biologia Animal, Biologia Vegetal i Ecologia, Unitat d’Antropologia Biològica Universitat Autònoma de Barcelona, Bellaterra - Cerdanyola del Valles, Spain; Serv. C.O.T. Hosp., Sagrat Cor/Unidad Docente U.B., Viladomat 288, 08029 Barcelona, Spain

**Keywords:** Ancient pathologies, Shoes, Egypt, Mummies

## Abstract

**Background:**

In ancient populations, a significant quantity of foot pathology was related either to the type of footwear they used or the underlying terrain they walked on. Our study was carried out to analyze these parameters with the foot pathologies the mummies presented.

**Methods:**

Between 2006 and 2012, more than 650 individuals were recovered from the Sharuna and Qarara necropolis (Middle Egypt) dating from the VIth Dynasty of the first Ptolemaic Period to the second Coptic Period. From among them, a total of 73 mummified feet (41 from Sharuna and 32 from Qarara) were studied. We took into account the differences existing between both sites in location (15 km apart) and in time (2500 years apart).

**Results:**

Almost all feet from Sharuna were wrapped and impregnated with a preservative substance (anthropological mummification), while the mummification process in Qarara was quite natural. Pathologies were found in 36 of the 73 ft (20 from Sharuna and 16 from Qarara). The differences in foot pathologies between the two sites were analysed.

**Conclusions:**

The foot pathologies we found in both necropolises have led us to hypothesise that the majority of the diachronic differences could be related more to progressive changes in the type of the terrain brought out through droughts, than the changes in footwear habits.

## Background

In ancient populations, apart from congenital abnormalities and tumours, a significant number of foot alterations and pathologies were related to the type of footwear and the nature of the terrain. Despite the importance of this relationship few studies have referenced types of foot pathologies in relation to their lifestyles in ancient times [1].

**Fig. 1:**
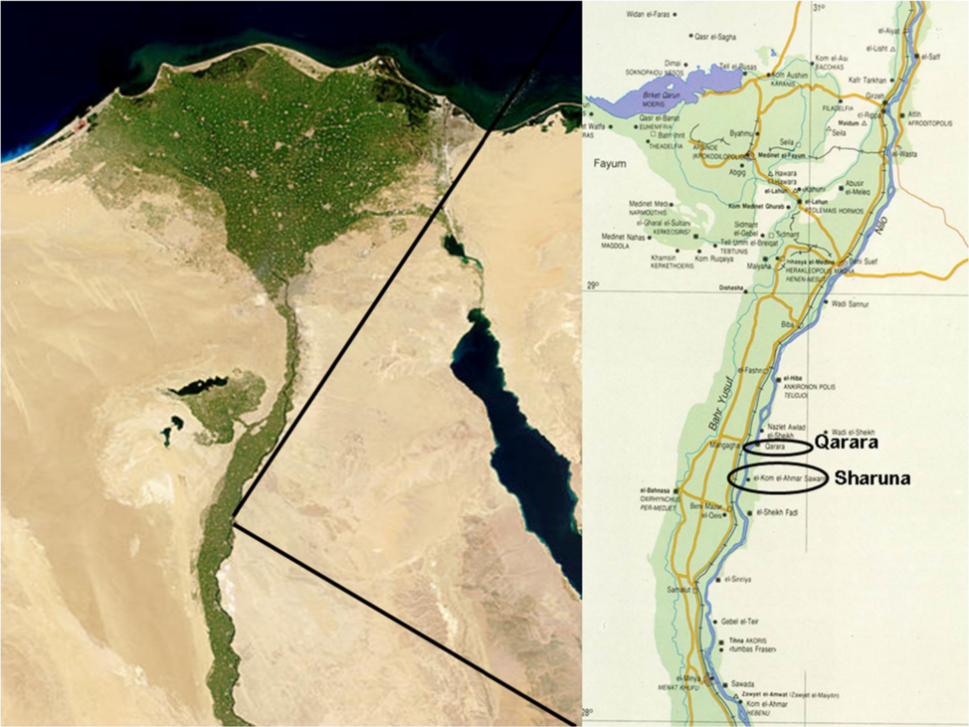
Map of the region. Enlarged map shows the North-Upper Egypt where the two sites are located

Between 2007 and 2012, archaeological teams from the *Museu Egipci* in Barcelona (Catalonia, Spain) and the *Äegyptolisches Institut* of the Eberhart-Karls University in Tübingen (Germany) collaborated on site at Sharuna and Qarara. [2] (Fig. 1). These archaeological sites have revealed more than 650 individuals to date. However, most human remains of these individuals were discovered dismembered, with poor anatomical association or even moved from the original burial site where they were interred by specific burial rituals [3].

Sharuna (S1) is a large Egyptian necropolis located on the east bank of the Nile River in Middle Egypt (about 200 km south of Cairo and 60 north of Minia). This site covers a wide range of periods between the 3rd Dynasty and the Coptic Period with the main anthropological site being the Tomb U.20. Wilkinson first mentioned this necropolis in 1835 and Nestor l’Hôte in 1838 had described the Tomb of the Pharaoh Pepi II of the 6th Dynasty as an important tomb at the site [4].

The Qarara (Q2) necropolis is located 15 Km north of Sharuna and is a huge burial area in which people from the Coptic Period were interred. Its time period ranges from the 5^th^ to the 14^th^ century AD. Although the two necropolises are not far each other there is a time difference of about 2500 years between them. In Qarara, most of the individuals were discovered in a partially or completely mummified state (near 75 %) different to Sharuna in which the majority of them were found as raw-bone (near 85 %).

The aim of this study is to compare the pathologies found in the feet of the mummified individuals between the two necropolises which had a short distance but large time span between them analysing in the basis of bioclimatic differences, the soil types and differences in footwear.

## Methods

The fact that most of the documented anthropological remains were been found outside their original settings (especially in S1), made it difficult for us to ascribe the retrieved individuals to a particular period. However, through some typical characteristics such as the type and form of bandages in which the mummies were wrapped, the presence or absence of nasal tamponade, and the amount of resins in the abdominal, thoracic and/or cranial cavities, we were able to estimate when the mummification procedure took place [5]. In most individuals, we did not find any abdominal wall in an acceptable enough condition to enable us to determine the existence, or not, of an incision that was used to extract the internal organs. These different types of incisions would have provided more information about the period to which the mummy belonged [6].

Nonetheless, the characteristics of the mummification process that we did find, allowed us to ascribe S1 specimens to be from the 6th Dynasty to First Intermediate Period (2323–2040 BC) [7]. The specimens coming from Q2 belonged to the First Coptic period (between the IVth to VIIIth centuries).

A total of 73 ft, 41 from S1 and 32 from Q2, belonging to a minimum of 69 individuals were studied. There was a significant presence of infant and juvenile feet with 9 being from S1 and 8 from Q2. With reference to the adult individuals, we found that those from Q2 were older than those from S1. The age range in S1 was 30–40 years versus 45–55 years in the in Q2 individuals. In both the necropolises with respect to adult individuals we found a slightly higher prevalence of males (30 in S1 and 22 in Q2).

The Q2 ft did not present any problems to study due to their natural mummification process. However, the feet belonging to the occasional dismembered mummies found in the S1 necropolis needed to be carefully unwrapped. This procedure consisted of progressively moistening the outermost bandages and moving inwards; sometimes this process could take several days due to the sheets being glued together with a resin-like mass. The most difficult dressing to remove was those in contact with the skin. In order to study the morphology, as well as the pathologies, some of partial specimens were taken to the laboratory at the archaeological site where macroscopic, microscopic (x 5) and photographic studies were performed. Unfortunately, it was impossible to carry out field X-rays and Ca14 analysis.

## Results

### Mummification

Upon initial examination, there were clear differences between the mummified feet found in S1 and those from Q2. At the end of the Old Kingdom and the First Intermediate Period in S1, a different mummification process had been employed, depending on the social status, with the use of resin-like substances. In the Coptic period in Q2, about 2500 years later, people were sometimes buried with preservative substances like salt and juniper berries within the inner clothing layers. In Q2, the majority of individuals present no signs of body treatment [8, 9]. Probably, between the Vth and the Xth century AD, the Coptic people in Egypt, abandoned the ancient mummification methods [6].

**Fig. 2:**
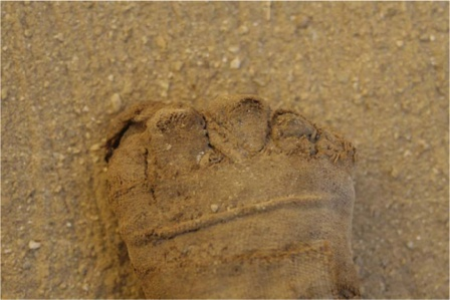
Detail of the children’s foot wrapping. Sharuna

**Fig. 3:**
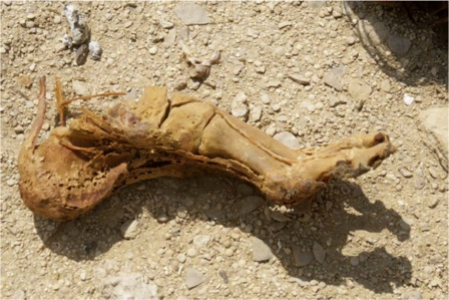
Partially mummified foot from Qarara. Tendons are well preserved

Almost all of the feet recovered from S1 were wrapped in linen bandages and impregnated with bitumen-like preservative substances. The big toe and the smaller toes were in some cases wrapped separately from each other, and then covered with an external bandage that included both feet. In some cases (mainly children) the skill and precision with which the bandages wrapped the feet are impressive (Fig. 2). After being unwrapped, the best preserved structures were the nails and the attached tendons. On the other hand, the preservation of feet from Q2, apart from the use of some natural preservatives, was due to the environment, through the dry and salty soil, low relative humidity and, in some cases exposure to air. In these individuals, it was common to find dried remains of muscle, tendons and skin (Fig. 3). In none of these individuals were sandals present on the feet. In the mummified children’s feet, the whole anatomical connection was maintained in many cases due to the good preservation of the capsular joints and tendons. In these cases signs of insect damage were very prevalent.

### Pathologies

The pathologies found at both sites were classified as osteochondritis, fractures, infections, arthropathies, entesopathies and some more specific alterations.

**Fig. 4:**
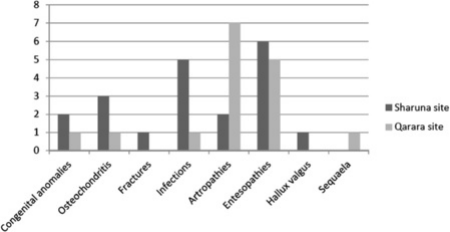
Distribution on feet pathologies present in Sharuna and Qarara

The 20 pathologies found in the Sharuna necropolis are shown in Fig. 4. There were three cases osteochondritis: two of them were in the central zone of the cartilage of the *acetabulum pedis* of the navicular bone and the last one on the distal articular surface of the proximal phalanx of the hallux (with no corresponding lesion on first metatarsal head). There was one fracture of the second metatarsal bone with no deviation of the diaphyseal line. Five bone infections were present: four were in diaphyseal bones (two in the proximal phalanges and two in metatarsal bones) and one in the cuboid. One case presented with hallux valgus (HV) with preservation of the capsular joint and sesamoid bones. There were two cases of Miller-Weiss syndrome (avascular necrosis of the navicular bone associated with talo-navicular arthropathy) and another two congenital abnormalities: a calcaneo-navicular synchondrosis and one massive tarsal coalition. And finally there were six cases of entesopathies in the calcaneus (Achilles and calcaneal spur).

**Fig. 5:**
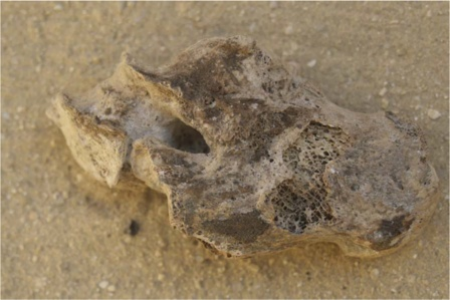
Massive tarsal coalition from Sharuna

**Fig. 6:**
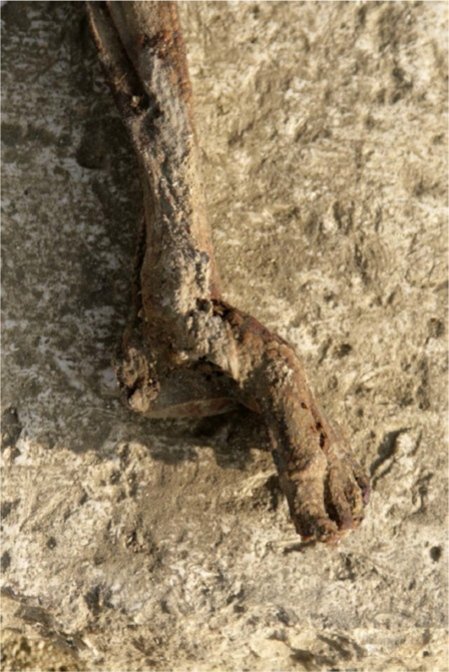
Talipes from Qarara. This case is not a post-mortem condition

The 16 pathologies found in the Qarara necropolis are shown in Fig. 4 and were classified as follows. There was one case of talar osteochondritis of the medial lip; one pseudocyst in the diaphysis of the 2nd metatarsal bone (of probable infectious origin from outside to inside); one massive tarsal coalition (Fig. 5); one case of talipes (this particular case was not due to a taphonomic or post-mortem cause when compared to all the other individuals buried in the same method) (Fig. 6); five cases of calcaneal spurs and seven cases of subtalar arthropathy (the most significant pathology in this area).

**Fig. 7:**
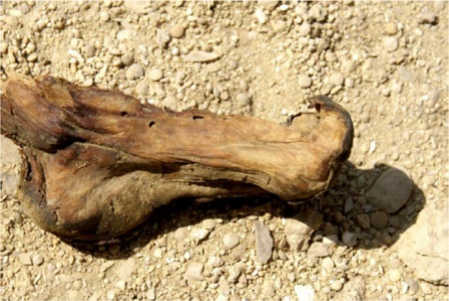
Effects of the wrap and the foot position after death mimicking a *hallux extensu*s. Qarara

Some cases of pseudo-pathology are present in both necropolises. It was very important not to confuse these conditions with diseases. From Q2, there is a clear case of a false hallux extensus in a complete mummified foot (Fig. 7) with a similar case present in a hallux from S1.

## Discussion

**Fig. 8:**
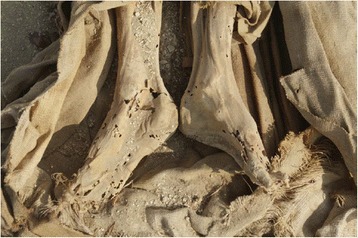
Natural mummified feet of a Coptic individual from Qarara necropolis. The holes are due at insect damage

In order to approximate a comprehensible picture of the health status in these two populations, we compared the pathologies present in the individuals of the two necropolises. The comparison allowed us to characterize the people and study their possible lifestyles and hence explore the sources of variations between them. In archaeological specimens, there is not always a clear cut border between those which are normal and those that are pathological. External agents can mimic abnormalities, either on the dry bone or in the mummified tissues. Sometimes these can be due to physical or chemical agents produced in the soil, sunlight, water, etc.; or by the direct action of living organisms such as plants (mainly roots) or animals (insects through bites and scratches) (Fig. 8). The position after death could have changed from the original burial one, either through natural or human actions. For instance, the preserved soft tissues of mummies could be in unusual positions which could be confused with deformities; and it is important to draw attention to the fact that these situations can also occur in our study. This was the case in the false hallux extensus above mentioned.

Taking into account all these considerations, a total of 36 ft with pathologies or related conditions were found among the 73 ft analysed, which represent 49.3 % of the sample. A similar percentage of pathologies were found in the two necropolises, but their distribution was different. Infections and osteochondritis were the most frequent pathologies found in Sharuna, while they are hardly present in Qarara. Conversely, entesopathies and arthropathies were common in the Qarara site and are scarce in Sharuna.

To explain these differences, we must bear in mind that most of the people buried in both necropolises belonged to the working class (workers in S1 and monks in Q2). Although humans used footwear (initially made of plant fibres or leather) in the Upper Palaeolithic period [10], it is probable that most of the exhumed individuals we studied walked barefoot all their lives (neither site had any specimen with footwear in the dressing). We believe that differences between the footwear habits of the inhabitants of both these areas are insufficient to explain the differences in pathologies found here. Sandals are known to have existed from the middle of the 3^rd^ millennium (Pyramids text) and in the middle of the 2^nd^ sandals were frequently used in Egypt, using wood and leather for soles [11]. At the end of the 6th Dynasty and the First Intermediate Period, shoes were made of hemp and linen and were totally flat, while in the early Coptic Period, shoes were also flat and made of leather [12]. We believe that the terrain and ground on which these people walked was more important than the footwear. The landscape in the Old Kingdom and First Intermediate Period was very different to what it is today, as the areas of alluvial soils were greater [13]. In the Coptic Period, the terrain had yet to change to one more or less similar to that of the present day, with sandy and rocky desert soils.

This terrain could shed some light on the high prevalence of degenerative subtalar arthropathy (7 of 21 more than 20 % of adult feet) in Q2, an alteration that could be associated with walking on irregular ground [14]. On the other hand, in S1, there were 5 notable cases of infection (12.2 %), a pathology that is absent in Q2, and which could be related to walking barefoot in marshes. In S1, there are 3 cases of osteochondritis in bones of the medial column of the foot (navicular and F1). It is remarkable that there is an almost complete absence of traumatic injuries (only 1 fracture in the neck of a second metatarsal bone - healed with a deviation of 30°) in both necropolises, a condition that could be related to walking barefoot [15]. It is also uncommon to find, in archaeological remains, 2 cases (1 in each necropolis) of tarsal coalition [16] although the lack of in-depth analysis, with radiological techniques for instance, means that we cannot rule out an ankylosing condition of non-congenital aetiology. The equine foot from Q2 is not the result of any post-mortem deformity, although it used to be a frequent condition in many mummified feet due either to muscular imbalance during the preservation process, or to external forces from bandaging and the sarcophagus [17]. Finally, in relation to the case of hallux valgus from S1, it is remarkable that in Ancient Egypt the bunion was not described in paleopathological literature, although diseases of the big toe must have been common. The importance that Egyptians attached to the big toe can be seen from its relevance in the art of the human figure, from the preservation of this part of the anatomy for the after-life and the presence of exo-prosthesis of the hallux in two mummies. The first case belonged to a female individual from the 21th Dynasty which consisted of a two-component hallux prosthesis of the right foot [18], and the second was a superbly crafted wooden prosthesis after hallux amputation in an individual from the early Third Intermediate Period (21th to 22th Dynasty) [19].

## Conclusions

The analysis of foot pathologies and their incidence in Sharuna and Qarara necropolises show the influence of environment and customs on the lives of these people. The majority of diachronic differences that we found appear more to be related to the type of terrain encountered than to their footwear habits.
